# Association of High-Volume Centers With Survival Outcomes Among Patients With Nontraumatic Out-of-Hospital Cardiac Arrest: A Systematic Review and Meta-Analysis

**DOI:** 10.1001/jamanetworkopen.2022.14639

**Published:** 2022-05-31

**Authors:** Amelia Xin Chun Goh, Jie Cong Seow, Melvin Yong Hao Lai, Nan Liu, Yi Man Goh, Marcus Eng Hock Ong, Shir Lynn Lim, Jamie Sin Ying Ho, Jun Wei Yeo, Andrew Fu Wah Ho

**Affiliations:** 1 Yong Loo Lin School of Medicine, National University of Singapore, Singapore; 2 Center for Quantitative Medicine, Duke-NUS (National University of Singapore) Medical School, Singapore; 3 Health Services and Systems Research, Duke-NUS Medical School, Singapore; 4 Department of Emergency Medicine, Singapore General Hospital, Singapore; 5 Department of Cardiology, National University Heart Center, Singapore; 6 Academic Foundation Programme, Royal Free London NHS (National Health Service) Foundation Trust, London, United Kingdom; 7 Prehospital and Emergency Research Center, Duke-NUS Medical School, Singapore

## Abstract

**Importance:**

Although high volume of cases of out-of-hospital cardiac arrest (OHCA) is a key feature of cardiac arrest centers, which have proven survival benefit, the role of center volume as an independent variable associated with improved outcomes is unclear.

**Objective:**

To assess the association of high-volume centers with survival and neurological outcomes in nontraumatic OHCA.

**Data Sources:**

Medline, Embase, and the Cochrane Central Register of Controlled Trials were searched from inception to October 11, 2021, for studies including adult patients with nontraumatic OHCA who were treated at high-volume vs non–high-volume centers.

**Study Selection:**

Randomized clinical trials, nonrandomized studies of interventions, prospective cohort studies, and retrospective cohort studies were selected that met the following criteria: (1) adult patients with OHCA of nontraumatic etiology, (2) comparison of high-volume with low-volume centers, (3) report of a volume-outcome association, and (4) report of outcomes of interest. At least 2 authors independently reviewed each article, blinded to each other’s decision.

**Data Extraction and Synthesis:**

Data abstraction and quality assessment were independently conducted by 2 authors. Meta-analyses were performed for adjusted odds ratios (aORs) and crude ORs using a random-effects model. This study followed the Preferred Reporting Items for Systematic Reviews and Meta-analyses (PRISMA) reporting guideline.

**Main Outcomes and Measures:**

Survival and good neurological outcomes according to the Cerebral Performance Categories Scale at hospital discharge or 30 days.

**Results:**

A total of 16 studies involving 82 769 patients were included. Five studies defined high volume as 40 or more cases of OHCA per year; 3 studies defined high volume as greater than 100 cases of OHCA per year. All other studies differed in definitions. Survival to discharge or 30 days improved with treatment at high-volume centers, regardless of whether aORs (1.28 [95% CI, 1.00-1.64]) or crude ORs (1.43 [95% CI, 1.09-1.87]) were pooled. There was no association between center volume and good neurological outcomes at 30 days or hospital discharge in patients with OHCA (aOR, 0.96 [95% CI, 0.77-1.20]).

**Conclusions and Relevance:**

In this meta-analysis and systematic review, care at high-volume centers was associated with improved survival outcomes, even after adjustment for potential confounders, but was not associated with improved neurological outcomes for patients with nontraumatic OHCA. More studies evaluating the relative importance of center volume compared with other variables (eg, the availability of treatment modalities) associated with survival outcomes in patients with OHCA are required.

## Key Points

**Question:**

Is treatment at a high-volume center associated with improved survival and neurological outcomes among adult patients with nontraumatic out-of-hospital cardiac arrest (OHCA)?

**Findings:**

In this systematic review and meta-analysis of 16 articles involving 82 769 patients with OHCA, survival to discharge or 30 days improved with treatment at a high-volume center; there was no association between center volume and good neurological outcomes at 30 days or at hospital discharge.

**Meaning:**

These findings suggest that treatment at a high-volume center may improve survival but not neurological outcomes in patients with OHCA; more studies evaluating the relative importance of center volume compared with other variables associated with survival outcomes in these patients are required.

## Teaser

This meta-analysis and systematic review assesses whether treatment at a high-volume center is associated with better survival and neurological outcomes in patients with nontraumatic out-of-hospital cardiac arrest (OHCA).

## Introduction

Out-of-hospital cardiac arrest (OHCA) is a time-critical medical emergency that results in substantial disease burden.^1,2^ Outcomes in OHCA can be poor despite the return of spontaneous circulation^3^ because post–cardiac arrest syndrome, a systemic ischemia-reperfusion injury, is a major contributor to mortality and morbidity in patients with OHCA.^4^ Consequently, post–cardiac arrest care has been advocated as the fifth link in the chain of survival.^5^ Considering the advanced treatment required, such as targeted temperature management (TTM) and percutaneous coronary intervention (PCI), specialized tertiary centers with access to such facilities are recommended to manage cases of OHCA.^6,7,8^

The association of regionalization of care to high-volume hospitals and improved outcomes has been observed in cardiological diseases and procedures, including cardiogenic shock and extracorporeal membrane oxygenation.^9,10^ Although there have been suggestions of such benefits in OHCA management, they have not been consistently observed. A refined understanding of the volume-outcome association in patients with OHCA aids policy recommendations on emergency transportation to improve the care of these patients.^11^ Furthermore, although high volume of cases of OHCA has been deemed a key feature of cardiac arrest centers (CACs),^7^ it is unclear whether case volume is independently associated with improved outcomes for patients with OHCA who are treated at CACs.^12^

We hypothesized that a high-volume center is associated with better clinical outcomes, namely survival to hospital discharge or 30 days and neurological outcomes at hospital discharge or 30 days among patients with OHCA. We performed a systematic review and meta-analysis to test this hypothesis.

## Methods

This systematic review and meta-analysis adhered to the Preferred Reporting Items for Systematic Reviews and Meta-analyses (PRISMA) reporting guideline. The study protocol has been published on the International Prospective Register of Systematic Reviews (PROSPERO identifier: CRD42022300967).

### Search Strategy

We performed a systematic literature search in Medline, Embase, and the Cochrane Central Register of Controlled Trials using a search strategy developed in consultation with a medical information specialist on October 11, 2021. To retrieve relevant articles, we used keywords and MeSH terms such as *hospital volume*, *patient volume*, *regionalisation*, *out-of-hospital cardiac arrest*, and other synonyms in the search strategy. We consulted content experts (M.E.H.O. and A.F.W.H.) for additional references and hand-searched bibliographies of relevant sources to identify additional relevant studies. We used EndNote, version X9 (Clarivate Analytics),^13^ to view and sieve articles. The search was repeated on January 1, 2022, which found no additional eligible articles. The detailed search strategy is available in the eMethods in the Supplement.

### Inclusion and Exclusion Criteria

Three authors (A.X.C.G., J.C.S., and M.Y.H.L.) sorted the retrieved articles using predefined criteria. At least 2 authors independently reviewed each article, blinded to each other’s decision. Disputes were resolved through consensus with a senior author (A.F.W.H.). The following inclusion criteria were used: (1) studies of adult patients with OHCA of nontraumatic etiology, (2) studies comparing high-volume centers with low-volume centers, (3) studies reporting a volume-outcome association, and (4) studies reporting outcomes of interest such as survival to hospital discharge or 30 days and good neurological outcomes at hospital discharge or 30 days. Cerebral Performance Categories Scale scores of 1 or 2 were considered a good neurological outcome, as defined by the studies included. The outcomes evaluated were at discharge and 30 days, because long-term outcomes were not reported in the literature. We included randomized clinical trials, nonrandomized studies of interventions, prospective cohort studies, and retrospective cohort studies. We excluded conference abstracts and reports without primary data such as reviews, meta-analyses, protocols, letters, commentaries, and editorials. We excluded studies with no control group or with only pediatric patients (<18 years of age) and non-English language studies without an English translation.

### Data Abstraction 

Data on general article information (author, year, and country), baseline demographic characteristics of patients (age, sex, and OHCA etiology), definition of high volume (annual volume of cases of OHCA), study location (emergency department, intensive care unit, or hospital), and outcomes of interest (survival and good neurological outcomes at hospital discharge or to 30 days) were abstracted by 3 authors (A.X.C.G., J.C.S., and M.Y.H.L.). The process of data abstraction was blinded among the authors, using a predesigned data abstraction form. Disputes were resolved through consensus with a senior author (A.F.W.H.). We also abstracted adjusted odds ratios (aORs) and crude ORs for binary outcomes from each article. For ORs adjusted using incremental or hierarchical statistical models, we abstracted the aOR for the final model presented. Where multiple statistical approaches were presented (eg, regression modeling and propensity score matching) in the same study, we considered the approach used in the primary analysis. When summary effect size estimates were unavailable, we calculated ORs and 95% CIs using summary data within 2 × 2 contingency tables if reported in the study.

### Statistical Analysis

We performed conventional pairwise meta-analyses comparing high-volume and low-volume centers. We preferentially analyzed aORs over ORs because aORs are less likely to be influenced by confounders. Both aORs and ORs were pooled and presented because they provide different insight on direct and indirect associations, respectively. We analyzed the estimate for the highest vs lowest volume (eg, quartile 1 vs quartile 4 for studies that split volume into quartiles; high vs low volume for studies that split volume into low, medium, and high) to identify the possible association of volume with outcomes. We applied a DerSimonian-Laird random-effects model with inverse variance weights owing to expected between-study variations in population and interventions. Heterogeneity was assessed using the *I*^2^ statistic with thresholds of 25% for low levels, 50% for moderate levels, and 75% for high levels. To account for heterogeneity, subgroup analyses were performed to compare studies defining high volume as 40 or more vs less than 40 cases of OHCA annually and also among all included studies for predefined, clinically important Utstein Formula of Survival variables^14^: initial shockable rhythm and presence of prehospital return of spontaneous circulation whenever possible. The cutoff of at least 40 cases of OHCA per year for high-volume centers was based on recommendations from the published 2020 Acute CardioVascular Care of the European Society of Cardiology (ACVC) position paper.^7^ To further explore any possible volume-outcome association, we performed a dose-response meta-analysis (DRMA) as a sensitivity analysis according to the method described by Berlin et al,^15^ using mean or median center volumes that were assigned to the corresponding natural logs of ORs or 95% CIs for each respective study arm. All analyses were performed using RevMan, version 5.4 (Cochrane Collaboration),^16^ and R, version 4.1.0 (R Core Team).^17^ Two-tailed statistical significance was set at *P* < .05. Publication bias was assessed through visually inspecting funnel plots when 10 or more studies reported an outcome. The quality of observational studies was evaluated on the Newcastle-Ottawa scale.

## Results

### Literature Retrieval and Summary of Included Articles

**Figure 1.**
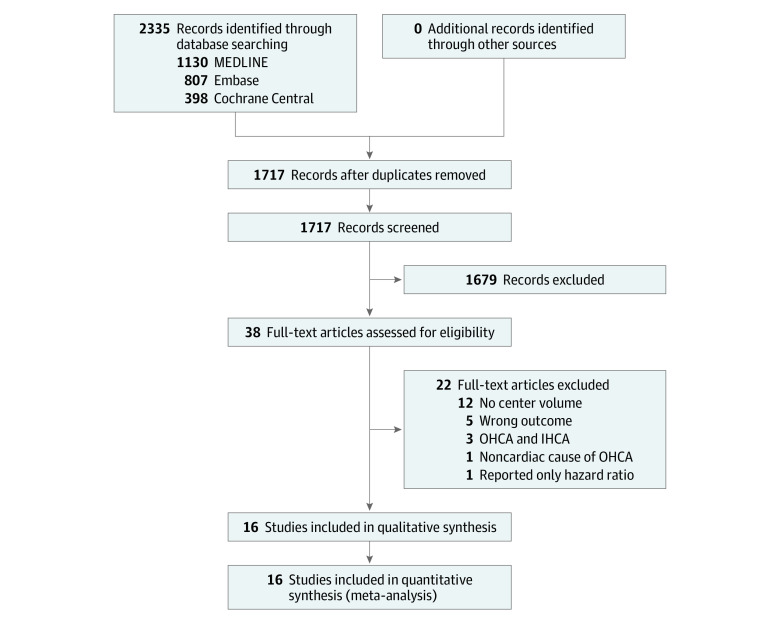
Study Selection Flowchart IHCA indicates in-hospital cardiac arrest; OHCA, out-of-hospital cardiac arrest.

The database search yielded 2335 articles. A total of 618 duplicated articles were removed; 1679 articles were excluded based on their titles and abstracts; and a further 22 articles were excluded on full-text review. Sixteen studies^18,19,20,21,22,23,24,25,26,27,28,29,30,31,32,33^ qualified for analysis. The study selection process and reasons for excluding the 22 studies are detailed in the flowchart in Figure 1. Interrater agreement was excellent (κ = 0.978).

A total of 82 769 patients were included in the 16 studies. One study was conducted in Austria,^29^ 1 in Australia,^33^ 1 in Canada,^33^ 1 in France,^21^ 2 in Japan,^24,26^ 4 in South Korea,^20,25,28,30^ 2 in the United Kingdom,^22,32^ and 4 in the US.^18,19,23,27^ Two studies^18,23^ reported data from the Cardiac Arrest Registry to Enhance Survival. Two studies^23,29^ included prospective cohorts and 14 studies^18,19,20,21,22,23,24,25,26,27,28,30,31,32,33^ included retrospective cohorts.

The characteristics and quality assessment of the included studies are presented in the eTable in the Supplement. The summary of meta-analysis results is presented in the Table.

**Table.** Summary of Meta-analysis Results

Outcome	No. of studies	Sample size	Effect size, OR (CI)	*P* value	*I*^2^ value, %
**Survival to discharge or 30 d**					
High-volume centers only					
Adjusted analyses	10	54 531	1.28 (1.00-1.64)	.05	85
Unadjusted analyses	12	55 477	1.43 (1.09-1.87)	.009	94
Subgroup analyses, ≥40 vs <40 cases of OHCA per year	12	58 917	1.08 (0.92-1.29) vs 1.58 (1.01-2.49	.13	NA
**Neurological outcomes at discharge or 30 d**					
High-volume centers only					
Adjusted analyses	9	32 944	0.96 (0.77-1.20)	.71	79
Unadjusted analyses	6	26 220	1.09 (0.88-1.35)	.42	81
Subgroup analyses, ≥40 vs <40 cases of OHCA per year	10	36 752	1.03 (0.76-1.41) vs 0.98 (0.72-1.33)	.81	NA

Abbreviations: OHCA, out-of-hospital cardiac arrest; OR, odds ratio.

### Definition of High Volume

The defining cutoff values of high-volume centers varied across studies. High volume was defined as 40 or more cases of OHCA per year in 5 studies^19,23,26,27,31^; more than 100 cases of OHCA per year in 3 studies^28,29,32^; more than 84 cases of OHCA within 5 years in 1 study^18^; more than 79 cases of OHCA within 15 months in 1 study^24^; more than 33 cases of OHCA per year in 1 study^20^; more than 25 cases of OHCA per year in 1 study^22^; more than 15 cases of OHCA per year in 1 study^21^; more than 25 cases of TTM per year in 1 study^33^; more than 15.5 cases of TTM per year in 1 study^25^; and more than 69 cases of cardiopulmonary resuscitation within 2 years in 1 study.^30^

The studies varied in how the defining cutoff was derived. Among the 5 studies that defined high volume as 40 or more cases of OHCA per year, 2 studies^23,26^ adapted the cutoff values from recent studies that evaluated the impact of volume on patients with OHCA, 1 study^27^ adopted the recommended annual volume of cases of OHCA proposed by the American Heart Association for CACs, 1 study^19^ plotted survival against the annual number of cases of OHCA in the database for all hospitals and found the highest survival rate in the group with 40 or more cases of OHCA per year, and 1 study^31^ provided no details on the derivation. Among the 3 studies that defined high volume as more than 100 cases of OHCA per year, 1 study^28^ adapted the definition from previous studies, whereas the remaining 2 studies^29,32^ did not describe how the definition was derived. The lone study that defined high volume as more than 84 cases of OHCA within 5 years derived this definition by patient data aggregation at the hospital level and calculation of the number of post–cardiac arrest episodes within each hospital.^18^ The lone study that defined high volume as more than 79 cases of OHCA within 15 months^24^ derived the definition by trisecting the total number of annual cases of OHCA equally into low-, medium-, and high-volume groups. The lone study that defined high volume as more than 33 cases of OHCA per year^20^ derived the definition from previous research in South Korea. The lone study that defined high volume as more than 25 cases of TTM per year^33^ based the definition on consensus among the investigators. The studies that defined high volume as more than 15 and more than 25 cases of OHCA per year^21,22^ did not explain the derivation. The studies that defined high volume as more than 15.5 cases of TTM per year^25^ and more than 69 cases of cardiopulmonary resuscitation within 2 years^30^ conducted sensitivity analysis using the area under the receiver operating characteristic curve.

### Survival to Hospital Discharge or 30 Days

#### Adjusted Analyses

**Figure 2.**
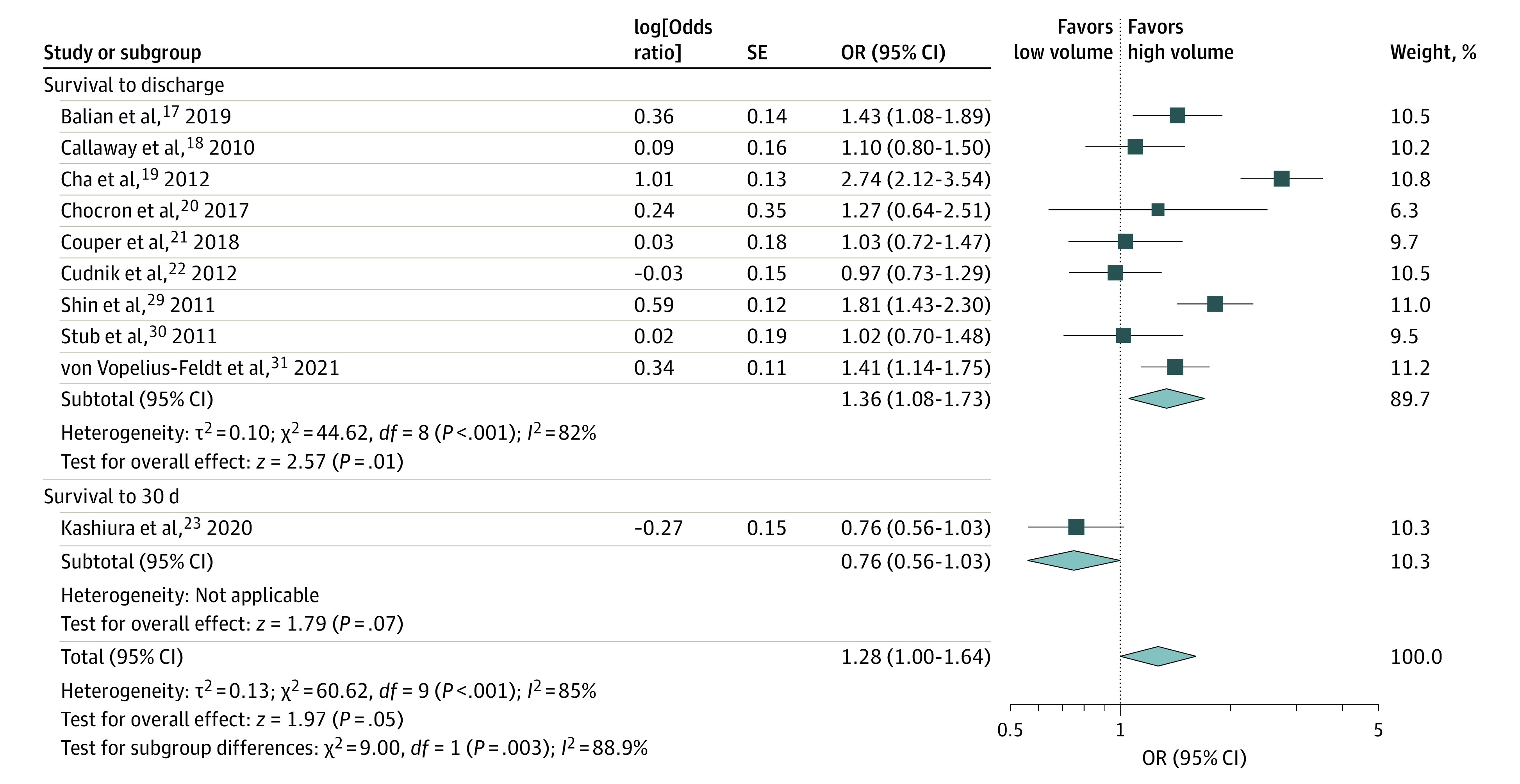
Adjusted Odds of Survival to Charge and to 30 Days OR indicates odds ratio. Different size markers account for weight.

Ten studies,^18,19,20,21,22,23,24,30,31,32^ which included 54 531 patients, reported aORs for survival to 30 days or at hospital discharge. Pooled analysis revealed an increase in survival among patients treated at high-volume centers (aOR, 1.28 [95% CI, 1.00-1.64]) (Figure 2). There was high between-study heterogeneity (*I^2^* = 85%).

#### Unadjusted Analyses

Eleven studies,^20,21,22,23,24,25,28,29,30,31,32^ which included 55 477 patients, reported crude ORs for survival to 30 days or at hospital discharge. Pooled analysis revealed a significant increase in survival among patients treated at high-volume centers (OR, 1.43 [95% CI, 1.09-1.87]). There was high between-study heterogeneity (*I^2^* = 94%).

### Neurological Outcomes at Hospital Discharge or 30 Days

#### Adjusted Analyses

**Figure 3.**
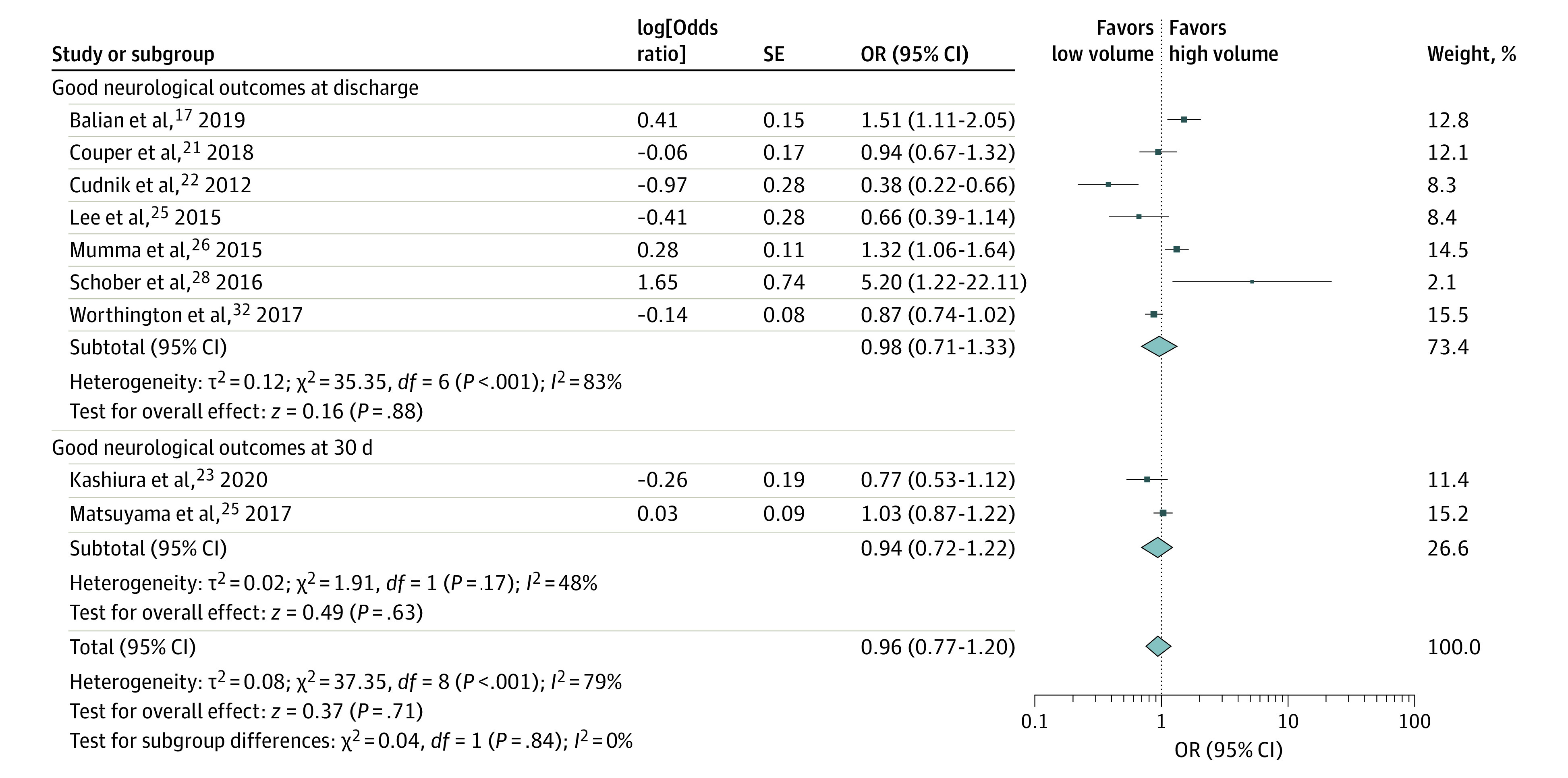
Adjusted Odds of Good Neurological Outcomes at Discharge and 30 Days OR indicates odds ratio. Different size markers account for weight.

Nine studies,^18,22,23,24,25,26,27,29,33^ which included 32 944 patients, reported aORs for good neurological outcomes at 30 days or at hospital discharge. Pooled analysis showed no significant difference in neurological outcomes among patients treated at high-volume centers (aOR, 0.96 [95% CI, 0.77-1.20]) (Figure 3). There was high between-study heterogeneity (*I^2^* = 79%).

#### Unadjusted Analyses

Six studies^18,24,25,26,28,33^ including 26 220 patients reported crude ORs for good neurological outcomes at 30 days or at hospital discharge. Pooled analysis revealed no significant difference in neurological outcomes among patients treated at high-volume centers (OR, 1.09 [95% CI, 0.88-1.35]). There was high between-study heterogeneity (*I^2^* = 81%).

### Subgroup Analysis of Studies With Cutoff of 40 or More vs Less Than 40 Cases of OHCA per Year

#### Survival to Hospital Discharge or 30 Days

There was no significant difference in survival to hospital discharge or 30 days between patients treated at centers with a cutoff value for high volume of 40 or more cases of OHCA per year^19,23,24,28,31,32^ and those treated at centers with a cutoff value for high volume of less than 40 cases of OHCA per year^18,20,21,22,25,30^ (χ^2^_1_ = 2.35; *P* = .13) (eFigure 1 in the Supplement). A DRMA found no significant association between center volume and survival to discharge or 30 days (*P* = .84) (eFigure 2 in the Supplement).

#### Neurological Outcomes at Hospital Discharge or 30 Days

There was no significant difference in neurological outcomes between patients treated at centers with a cutoff value for high volume of 40 or more cases of OHCA per year^23,24,26,27,28,29^ and those treated at centers with a cutoff value for high volume of less than 40 cases of OHCA per year^18,22,25^ (χ^2^_1_ = 0.06; *P* = .81) (eFigure 1 in the Supplement). A DRMA found no significant association between center volume and neurological outcomes at discharge or 30 days (*P* = .78) (eFigure 2 in the Supplement).

## Discussion

To our knowledge, this is the first systematic review and meta-analysis on the association of treatment at high-volume centers with the outcomes of patients with OHCA. The main results suggest that patients with OHCA treated at high-volume centers have improved survival compared with patients treated at low-volume centers. This survival benefit was attenuated, but remained resilient, after aORs were pooled. However, there was no association between center volume and neurological outcomes in patients with OHCA. A DRMA did not detect a dose-response association between survival or neurological outcomes at discharge or 30 days.

Regionalization of care is a proven approach in areas such as major trauma care and coronary artery disease.^7^ The success of this approach has been attributed to increased familiarity of procedures,^34^ experienced personnel, and well-established protocols.^35^ However, evidence for regionalization in cardiac arrest care emerged more recently and has inconclusive benefits.^12^ The recent 2020 ACVC position paper recommended the regionalization of patients with OHCA in CACs if local facilities are unable to deliver comprehensive post–cardiac arrest care.^7^ Although the definition of CACs varies widely, it is often understood as having high annual OHCA volume and the capability to deliver a bundle of interventions.^8^ Our finding of improved survival in high-volume centers supports this recommendation. However, closer inspection of the studies that reported aORs revealed that PCI, extracorporeal membrane oxygenation, and TTM capabilities were not adjusted for by most studies. Hence, the survival benefit of high annual OHCA volume may have been confounded by the aforementioned factors. For example, Callaway et al^19^ found that PCI capability and center volume of 40 or more annual cases of OHCA resulted in higher survival, but none were independent factors in determining survival.

Interestingly, we did not find evidence of better neurological outcomes in patients with OHCA treated at high-volume centers. The narrow final pooled 95% CIs further suggest that even if an association was found, as in the case of survival to discharge and survival to 30 days, it would unlikely be a large one. Given that a previous meta-analysis by Yeo et al^8^ found better survival and neurological outcomes in patients with OHCA treated at CACs, volume may not be directly associated with survival, and other components in the CAC bundle of interventions such as the availability of 24/7 access to PCI, TTM, and protocolized care in the intensive care unit contribute more to the benefit of CACs. This may be supported by the finding by Yeo et al^8^ that the effect of CACs, while significant, was attenuated when a sensitivity analysis with high-volume centers only was conducted. Instead of volume-based regionalization, it may be prudent for the regionalization of cardiac arrest care to focus on other aspects of postresuscitation care such as the availability of advanced treatment modalities, structured algorithms of care, and rehabilitation. Future studies investigating the effect of high-volume centers on patients with OHCA may consider adjusting for other components of post–cardiac arrest care.

Contrary to the statement made by the ACVC in their 2020 position paper that treatment of at least 40 patients with OHCA per year was associated with improved outcomes, we did not find significant subgroup differences across both survival and neurological outcomes between studies that defined high volume as 40 or more vs less than 40 cases of OHCA per year. This may be because the cutoff value was based on a 2010 study by Callaway et al,^19^ who derived the value by plotting survival against the annual number of cases of OHCA treated by each hospital, although their study was not intended or designed to study threshold effects and to recommend a threshold. Therefore, caution should be exercised when adopting the cutoff of 40 or more annual cases of OHCA. Future studies may consider using standardized methods to determine individualized cutoff volumes, which can then be meta-analyzed to derive a universal cutoff value.

The DRMA found a lack of dose-response association between center volume and survival or neurological outcomes in patients with OHCA. This may be due to the inclusion of studies in which participating centers had access to the aforementioned bundle of interventions, regardless of center volume, because low volume does not mean limited resources.^24^ It is also possible that insufficient statistical power had prevented the detection of a dose-response association, even if one was present. Finally, our DRMA can only be interpreted for the range of dosage represented, and findings cannot be extrapolated beyond this range.

Overall, we found that treatment at high-volume centers was associated with better survival outcomes but not neurological outcomes, which is becoming relatively important in cardiac arrest care compared with survival alone.^36^ Although our findings certainly do not support depriving patients with OHCA of care at CACs, it is important to highlight the potential pitfalls of using high volume as a key factor in deciding where patients with OHCA should be transported, as well as adopting 40 or more annual cases of OHCA as a cutoff for high volume.

### Strengths and Limitations

To our knowledge, this is the first systematic review and meta-analysis to assess the benefits of high-volume centers in the treatment of OHCA involving OHCA registries and databases from various nations and a large sample of 82 769 patients. Although the inclusion of studies from various geographical locations may increase the generalizability of our findings, it may have led to the high statistical heterogeneity. The inclusion of studies with varying definitions of high volume and approaches to analyzing volume may also have contributed to the high clinical heterogeneity. All included studies were nonrandomized studies of intervention, which are inherently susceptible to selection and observation biases. High-quality randomized clinical trials are needed to confirm the present findings, although this may be ethically challenging. Although we reduced the effect of confounding through pooling estimates from adjusted analysis, there remains the possibility of residual confounding arising from individual studies. Insufficient studies performed subgroup analysis according to prehospital Utstein Formula of Survival variables^37,38^ such as initial shockable rhythm or prehospital return of spontaneous circulation, which precluded subgroup analyses to determine differences in OHCA outcomes in different subpopulations treated at high-volume centers. Long-term neurological and functional outcomes were not reported in the literature and therefore could not be assessed.

## Conclusions

In this meta-analysis and systematic review, treatment of patients with OHCA at a high-volume center was associated with improved survival but not improved neurological outcomes at hospital discharge or 30 days. More high-quality studies are needed to evaluate the relative importance of center volume compared with other variables in post–cardiac arrest care such as PCI and TTM as an independent variable associated with survival outcomes in patients with OHCA. Future studies should also determine the volume range at which a measurable effect on survival or neurological outcomes can be observed.

## Notes

Supplement. **eMethods.** Detailed Search Strategy **eTable.** Summary of Included Studies **eFigure 1.** Subgroup Analysis of Studies With Cutoff ≥40 vs <40 Cases of OHCA per Year **eFigure 2.** Sensitivity Analysis With a Dose-Response Meta-analysis of Logarithmic Odds Ratios Against Mean Hospital Volume
eReferences
