# Supplementary material for: Association of High-Volume Centers With Survival Outcomes Among Patients With Nontraumatic Out-of-Hospital Cardiac Arrest: A Systematic Review and Meta-Analysis
Source: JAMA Netw Open. 2022 May 31;5(5):e2214639. doi: 10.1001/jamanetworkopen.2022.14639 (PMC9157264; doi:10.1001/jamanetworkopen.2022.14639)
Supplement: Supplement. — eMethods. Detailed Search Strategy eTable. Summary of Included Studies eFigure 1. Subgroup Analysis of Studies With Cutoff ≥40 vs <40 Cases of OHCA per Year eFigure 2. Sensitivity Analysis With a Dose-Response Meta-analysis of Logarithmic Odds Ratios Against Mean Hospital Volume eReferences [file jamanetwopen-e2214639-s001.pdf]

Supplementary Online Content

Goh AXC, Seow JC, Lai MYH, et al. Association of high-volume centers with survival outcomes among patients with nontraumatic out-of-hospital cardiac arrest: a systematic review and meta-analysis. *JAMA Netw Open*. 2022;5(5):e2214639. doi:10.1001/jamanetworkopen.2022.14639

eMethods. Detailed Search Strategy
eTable. Summary of Included Studies
eFigure 1. Subgroup Analysis of Studies With Cutoff ≥40 vs <40 Cases of OHCA per Year
eFigure 2. Sensitivity Analysis With a Dose-Response Meta-analysis of Logarithmic Odds Ratios Against Mean Hospital Volume
eReferences

This supplementary material has been provided by the authors to give readers additional information about their work.

© 2022 Goh AXC et al. *JAMA Network Open*.

eMethods. Detailed Search Strategy

Search strategy for Medline

1. Regional Medical Programs/
2. (regionali* or (regionali* adj3 care)).ab,kf,ti.
3. (regional system* or network or hospital volume or patient volume or case volume or disease volume).ab,kf,ti.
4. (volume adj3 (emergency department or ICU or intensive care or critical care)).ab,kf,ti.
5. (low-volume or high-volume).ab,kf,ti.
6. (central* adj3 (care or service* or program* or treatment)).ab,kf,ti.
7. Cardiac Arrest Registry.ab,kf,ti.
8. or/1-7
9. exp heart arrest/
10. cardiopulmonary resuscitation/ or advanced cardiac life support/
11. Out of Hospital Cardiac Arrest.ab,kf,ti.
12. OHCA.ab,kf,ti.
13. return of spontaneous circulation.ab,kf,ti.
14. ROSC.ab,kf,ti.
15. ((heart or cardiac or cardiovascular) adj1 arrest).ab,kf,ti.
16. asystole.ab,kf,ti.
17. pulseless electrical activity.ab,kf,ti.
18. Advanced Cardiac Life Support.ab,kf,ti.
19. ACLS.ab,kf,ti.
20. Ventricular Fibrillation/
21. (cardiopulmonary arrest or cardiopulmonary resuscitation).ab,kf,ti.
22. (cardio-pulmonary arrest or cardio-pulmonary resuscitation or CPR).ab,kf,ti.
23. code blue.ab,kf,ti.
24. or/9-23
25. 8 and 24
26. exp Organ Transplantation/ or "transplant*".ab,kf,ti.
27. 25 not 26
28. Animals/ not (Animals/ and Humans/)
29. 27 not 28
30. (exp Pediatrics/ or exp CHILD/) not exp Adult/
31. 29 not 30
32. (letter or comment or editorial or note or news).pt.
33. 31 not 32
34. Case Reports/ or (case report or case series).ti.
35. 33 not 34

Search strategy for Embase

1. 'regional medical programs'/de
2. regionali*:ab,ti,kw
3. (regionali* NEAR/3 care):ab,ti,kw
4. 'regional system*':ab,ti,kw
5. 'regional network*':ab,ti,kw
6. 'hospital volume':ab,ti,kw OR 'patient volume':ab,ti,kw OR 'case volume':ab,ti,kw OR 'disease volume':ab,ti,kw
7. (volume NEAR/3 'emergency department'):ab,ti,kw
8. (volume NEAR/3 'intensive care'):ab,ti,kw

© 2022 Goh AXC et al. *JAMA Network Open*.

9. (volume NEAR/3 'icu'):ab,ti,kw
10. (volume NEAR/3 'critical care'):ab,ti,kw
11. 'low volume':ab,ti,kw
12. 'high volume':ab,ti,kw
13. (central* NEAR/3 care):ab,ti,kw
14. (central* NEAR/3 service*):ab,ti,kw
15. (central* NEAR/3 program*):ab,ti,kw
16. (central* NEAR/3 treatment):ab,ti,kw
17. 'cardiac arrest registry':ab,ti,kw
18. #1 OR #2 OR #3 OR #4 OR #5 OR #6 OR #7 OR #8 OR #9 OR #10 OR #11 OR #12 OR #13 OR #14 OR #15 OR #16 OR #17
19. 'heart arrest'/exp
20. 'cardiac life support':ab,ti,kw
21. 'Ohca':ab,ti,kw
22. 'return of spontaneous circulation'/de
23. ((heart OR cardiac OR cardiovascular) NEAR/1 arrest):ab,ti,kw
24. Asystole:ab,ti,kw
25. 'pulseless electrical activity':ab,ti,kw
26. acls:ab,ti,kw
27. 'heart ventricle fibrillation'/de
28. 'cardiopulmonary arrest':ab,ti,kw OR 'cardiopulmonary resuscitation':ab,ti,kw
29. 'cardio-pulmonary arrest':ab,ti,kw OR 'cardio-pulmonary resuscitation':ab,ti,kw OR 'cpr':ab,ti,kw
30. code blue':ab,ti,kw
31. #19 OR #20 OR #21 OR #22 OR #23 OR #24 OR #25 OR #26 OR #27 OR #28 OR #29 OR #30
32. #18 AND #31
33. 'organ transplantation'/exp OR 'transplant*':ab,ti,kw
34. #32 NOT #33
35. 'animal'/exp NOT ('animal'/exp AND 'human'/exp)
36. #34 NOT #35
37. ('pediatrics'/exp OR 'child'/exp) NOT 'adult'/de
38. #36 NOT #37
39. 'article'/it
40. #38 AND #39
41. 'case report'/de OR 'case study'/de OR 'case study':ti OR 'case series':ti
42. #40 NOT #41

Search strategy for Cochrane CENTRAL

1. MeSH descriptor: [Regional Medical Programs] this term only
2. regionali*:ti,ab,kw
3. (regionali* NEAR/3 care):ti,ab,kw
4. ("regional system*" or "regional network" or "hospital volume" or "case volume" or "disease volume"):ti,ab,kw
5. (volume NEAR/3 (emergency department or ICU or intensive care or critical care)):ti,ab,kw
6. (low-volume or high-volume):ti,ab,kw
7. (central* NEAR/3 (care or service* or program* or treatment)):ti,ab,kw
8. (Cardiac Arrest Registry):ti,ab,kw
9. MeSH descriptor: [Heart Arrest] explode all trees
10. MeSH descriptor: [Cardiopulmonary Resuscitation] this term only
11. MeSH descriptor: [Advanced Cardiac Life Support] this term only
12. (Out of Hospital Cardiac Arrest):ti,ab,kw
13. OHCA:ti,ab,kw
14. (return of spontaneous circulation):ti,ab,kw
15. ROSC:ti,ab,kw
16. ((heart or cardiac or cardiovascular) NEAR/1 arrest):ti,ab,kw

© 2022 Goh AXC et al. *JAMA Network Open*.

17. Asystole:ti,ab,kw
18. (pulseless electrical activity):ti,ab,kw
19. ACLS:ti,ab,kw
20. MeSH descriptor: [Ventricular Fibrillation] this term only
21. (cardiopulmonary arrest or cardiopulmonary resuscitation):ti,ab,kw
22. (cardio-pulmonary arrest or cardio-pulmonary resuscitation or CPR):ti,ab,kw
23. (code blue):ti,ab,kw
24. #1 or #2 or #3 or #4 or #5 or #6 or #7 or #8
25. #9 or #10 or #11 or #12 or #13 or #14 or #15 or #16 or #1y or #18 or #19 or #20 or #21 or #22 or #23
26. #24 and #25
27. MeSH descriptor: [Organ Transplantation] explode all trees
28. #27 or transplant:ti,ab,kw
29. #26 not #28
30. MeSH descriptor: [Animals] this term only
31. MeSH descriptor: [Humans] this term only
32. #30 not (#30 and #31)
33. #29 not #32
34. MeSH descriptor: [Pediatrics] explode all trees
35. MeSH descriptor: [Child] explode all trees
36. MeSH descriptor: [Adult] explode all trees
37. (#34 or #35) not #36
38. #33 not #37
39. (article):pt
40. #38 and #39
41. (case report):ti or (case series):ti
42. #40 not #41

© 2022 Goh AXC et al. *JAMA Network Open*.

eTable. Summary of Included Studies

Author	*Country*	*Study Location*	*Comparison*	*Sample size*	*Age (mean SD)*	*Male*	*Definition of High Volume*	*NOS*
Balian 2019[1]	USA	Hospital	High volume centres	1453	65 (23)*	867	>84 OHCA cases/5 years	7
			Low volume centres	577	64 (22)*	371		
Callaway 2010[2]	USA	Hospital	Not stated	4087	Not stated		≥40 OHCA cases/year	9
Cha 2012[3]	South Korea	ED	High volume centres	11777	65 (51-75)*	7742	>33 OHCA cases/year	7
			Low volume centres	15885	66 (53-76)*	10438		
Chocron 2017[4]	France	Hospital	High volume centres	917	60.1 +/- 15.4	658	>15 OHCA cases/year	7
			Low volume centres	91	66.6 +/- 16.6	56		
Couper 2018[5]	UK	Hospital	High volume centres	635	Not stated		>25 OHCA cases/year	9
			Low volume centres	2422				
Cudnik 2012[6]	USA	ED	High volume centres	928	62.6	566	≥40 OHCA cases/year	7
			Low volume centres	698	63.7	397		
Kashiura 2020[7]	Japan	Hospital	High volume centres	912	71 (60-81)*	577	>79 OHCA/15 months	8
			Low volume centres	889	71 (60-82)*	575		
Lee 2015[8]	South Korea	ED	High volume centres	289	58 (47-70)*	201	>15.5 TTM cases/year	7
			Low volume centres	289	58 (48-69.5)*	200		
Matsuyama 2017[9]	Japan	Hospital	High volume centres	12932	Not stated		≥40 OHCA cases/year	8
			Low volume centres	3372				

© 2022 Goh AXC et al. *JAMA Network Open*.

Mumma 2015[10]	USA	Hospital	High volume centres	3340	65 (53-77)*	1956	≥40 OHCA cases/year	7
			Low volume centres	1862	69 (56-80)*	1120		
Park 2019[11]	South Korea	Hospital	High volume centres	1200	Not stated	804	>100 OHCA cases/year	7
			Low volume centres	2608		1760		
Schober 2016[12]	Austria	Hospital	High volume centres	378	60 (49-70)*	276	>100 OHCA cases/year	8
			Low volume centres	269	66 (52-75)*	181		
Shin 2011[13]	South Korea	ED	High volume centres	3533	60.9 +/- 19.4	2270	>69 CPR cases/2 years	7
			Low volume centres	3533	60.5 +/- 18.6	2322		
Stub 2011[14]	Australia	Hospital	High volume centres	1384	Not stated		≥40 OHCA cases/year	7
			Low volume centres	252				
Vopelius-Feldt 2021[15]	UK	Hospital	High volume centres	2279	73 (60-82)*	1503	>100 OHCA cases/year	7
			Low volume centres	2279	73 (61-82)*	1495		
Worthington 2017[16]	Canada	Hospital	High volume centres	978	64 +/- 16	674	>25 TTM cases/year	9
			Low volume centres	721	65 +/- 15	510		

NOS: Newcastle-Ottawa score; OHCA: out of hospital cardiac arrest; TTM: targeted temperature management
*Median, IQR

© 2022 Goh AXC et al. *JAMA Network Open*.

eFigure 1. Subgroup Analysis of Studies With Cutoff ≥40 vs <40 Cases of OHCA per Year

Survival to hospital discharge or 30 days

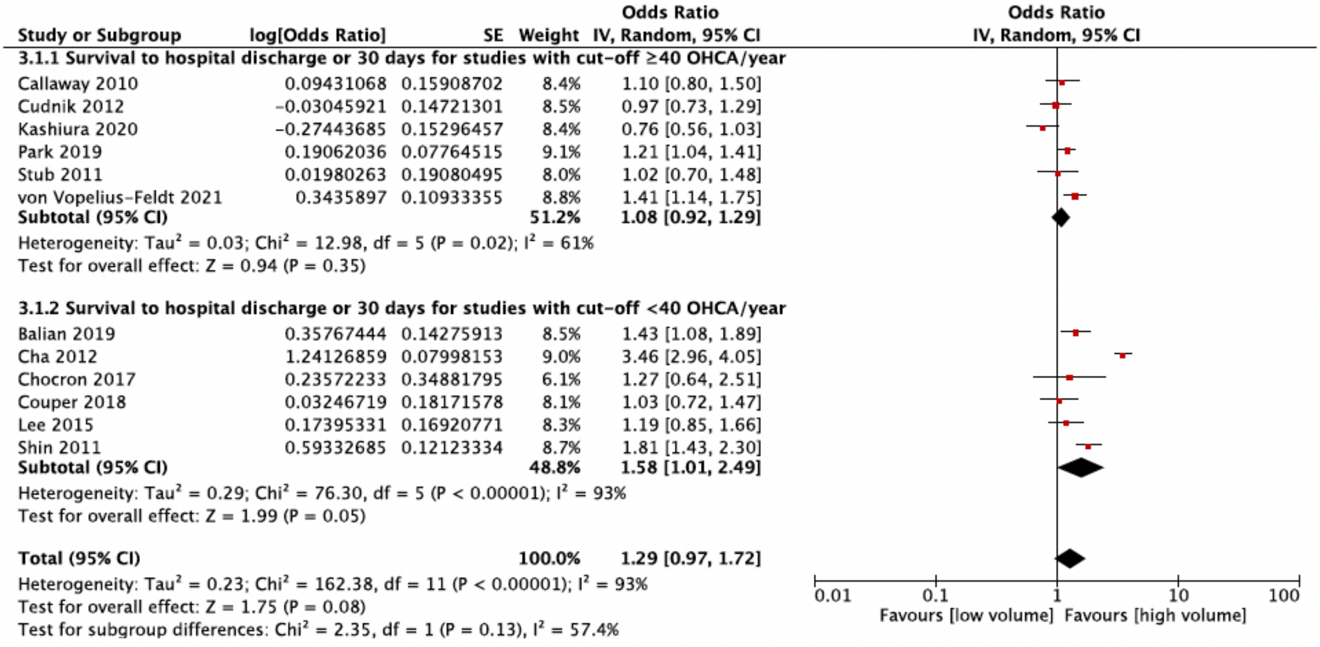

Neurological outcomes at hospital discharge or 30 days

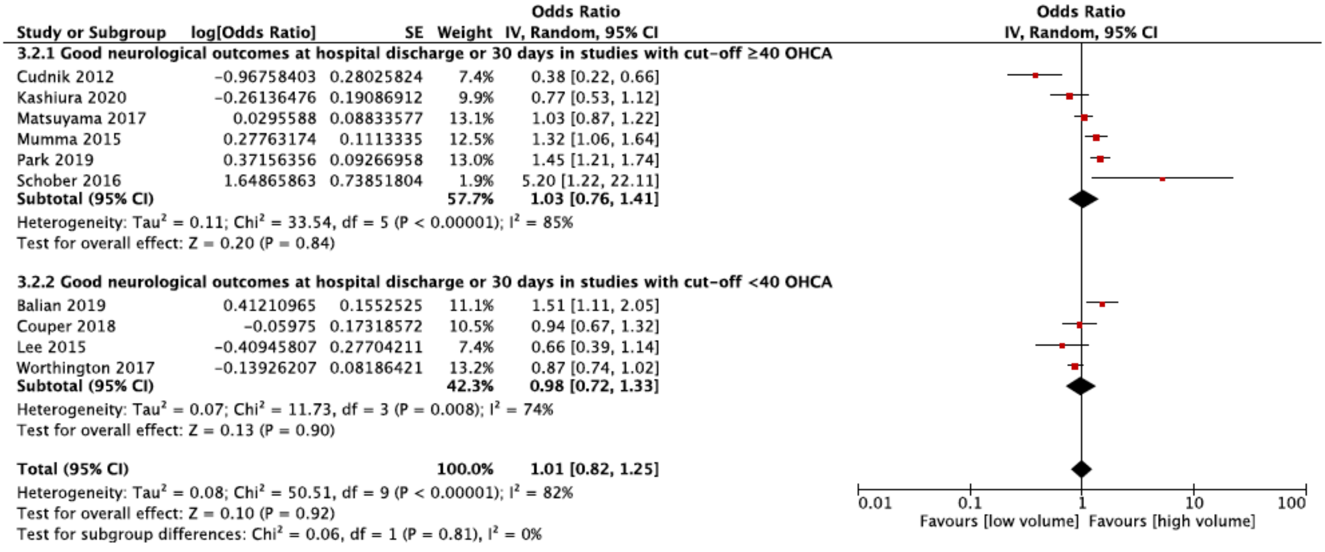

© 2022 Goh AXC et al. *JAMA Network Open*.

eFigure 2. Sensitivity Analysis With a Dose-Response Meta-analysis of Logarithmic Odds Ratios Against Mean Hospital Volume

Survival to hospital discharge or 30 days

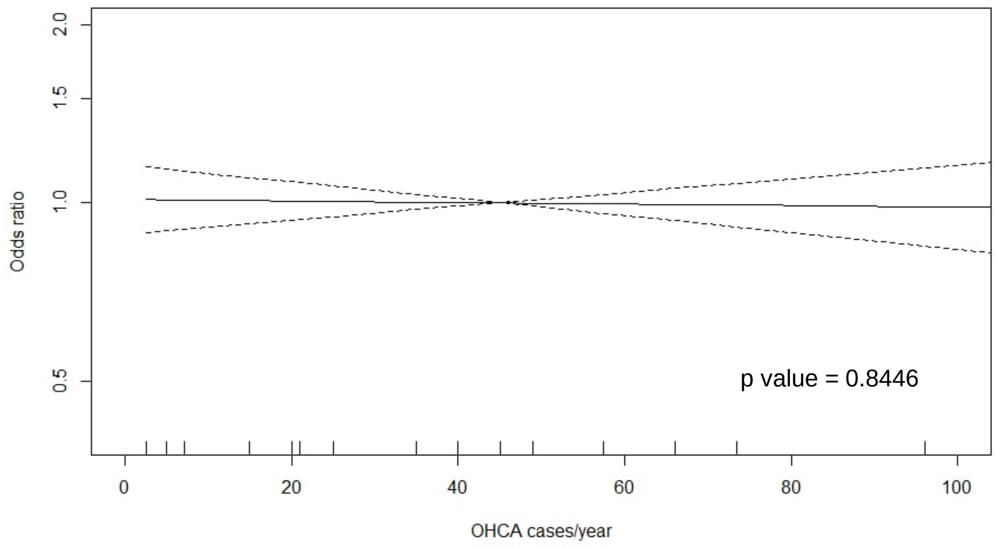

Survival to hospital discharge or 30 days with good neurological outcome

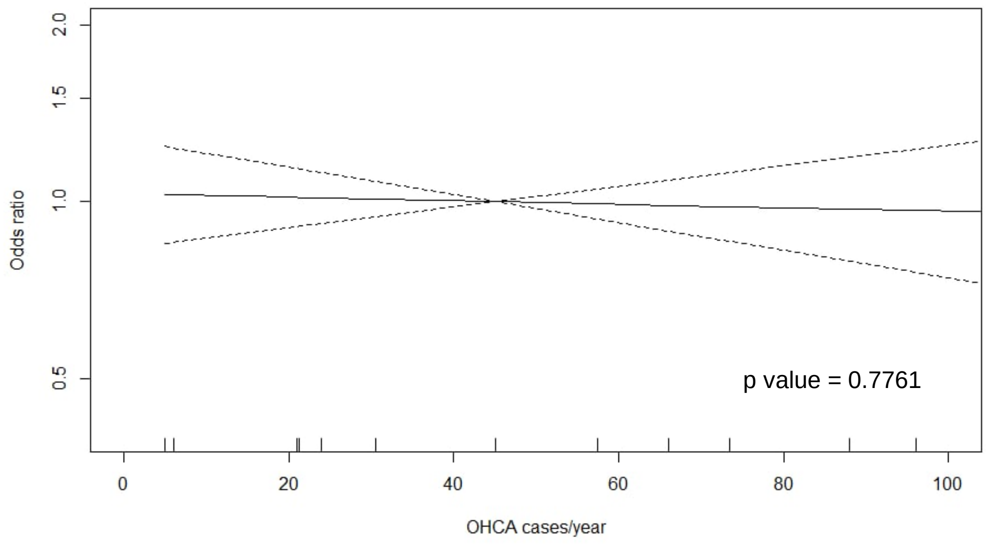

© 2022 Goh AXC et al. *JAMA Network Open*.

eReferences

1. Balian S, Buckler DG, Blewer AL, Bhardwaj A, Abella BS; CARES Surveillance Group. Variability in survival and post–cardiac arrest care following successful resuscitation from out-of-hospital cardiac arrest. Resuscitation. 2019;137:78-86.

2. Callaway CW, Schmicker R, Kampmeyer M, et al; Resuscitation Outcomes Consortium (ROC) Investigators. Receiving hospital characteristics associated with survival after out-of-hospital cardiac arrest. Resuscitation. 2010;81(5):524-529.

3. Cha WC, Lee SC, Shin SD, Song KJ, Sung AJ, Hwang SS. Regionalisation of out-of-hospital cardiac arrest care for patients without prehospital return of spontaneous circulation. Resuscitation. 2012;83(11):1338-1342.

4. Chocron R, Bougouin W, Beganton F, et al. Are characteristics of hospitals associated with outcome after cardiac arrest? insights from the Great Paris registry. Resuscitation. 2017;118:63-69.

5. Couper K, Kimani PK, Gale CP, et al. Variation in outcome of hospitalised patients with out-of-hospital cardiac arrest from acute coronary syndrome: a cohort study. NIHR Journals Library. 2018. Accessed December 2, 2021. https://www.ncbi.nlm.nih.gov/books/NBK488090/

6. Cudnik MT, Sasson C, Rea TD, et al. Increasing hospital volume is not associated with improved survival in out of hospital cardiac arrest of cardiac etiology. Resuscitation. 2012;83(7):862-868.

7. Kashiura M, Amagasa S, Moriya T, et al; SOS-KANTO 2012 Study Group. Relationship between institutional volume of out-of-hospital cardiac arrest cases and 1-month neurologic outcomes: a post hoc analysis of a prospective observational study. J Emerg Med. 2020;59(2):227-237.

© 2022 Goh AXC et al. *JAMA Network Open*.

8. Lee SJ, Jeung KW, Lee BK, et al; Korean Hypothermia Network (KorHN) Investigators. Impact of case volume on outcome and performance of targeted temperature management in out-of-hospital cardiac arrest survivors. Am J Emerg Med. 2015;33(1):31-36.
9. Matsuyama T, Kiyohara K, Kitamura T, et al. Hospital characteristics and favourable neurological outcome among patients with out-of-hospital cardiac arrest in Osaka, Japan. Resuscitation. 2017;110:146-153.
10. Mumma BE, Diercks DB, Wilson MD, Holmes JF. Association between treatment at an ST-segment elevation myocardial infarction center and neurologic recovery after out-of-hospital cardiac arrest. Am Heart J. 2015;170(3):516-523.
11. Park JH, Lee SC, Shin SD, Song KJ, Hong KJ, Ro YS. Interhospital transfer in low-volume and high-volume emergency departments and survival outcomes after out-of-hospital cardiac arrest: a nationwide observational study and propensity score–matched analysis. Resuscitation. 2019;139:41-48.
12. Schober A, Sterz F, Laggner AN, et al. Admission of out-of-hospital cardiac arrest victims to a high volume cardiac arrest center is linked to improved outcome. Resuscitation. 2016;106:42-48.
13. Shin SD, Suh GJ, Ahn KO, Song KJ. Cardiopulmonary resuscitation outcome of out-of-hospital cardiac arrest in low-volume versus high-volume emergency departments: an observational study and propensity score matching analysis. Resuscitation. 2011;82(1):32-39.
14. Stub D, Smith K, Bray JE, Bernard S, Duffy SJ, Kaye DM. Hospital characteristics are associated with patient outcomes following out-of-hospital cardiac arrest. Heart. 2011;97(18):1489-1494.

© 2022 Goh AXC et al. *JAMA Network Open*.

15. von Vopelius-Feldt J, Perkins GD, Benger J. Association between admission to a cardiac arrest centre and survival to hospital discharge for adults following out-of-hospital cardiac arrest: a multi-centre observational study. Resuscitation. 2021;160:118-125.

16. Worthington H, Pickett W, Morrison LJ, et al; Rescu Investigators. The impact of hospital experience with out-of-hospital cardiac arrest patients on post cardiac arrest care. Resuscitation. 2017;110:169-175.

© 2022 Goh AXC et al. *JAMA Network Open*.
